# Significance of distance between tumor and thyroid capsule as an indicator for central lymph node metastasis in clinically node negative papillary thyroid carcinoma patients

**DOI:** 10.1371/journal.pone.0200166

**Published:** 2018-07-17

**Authors:** Chan Yong Seong, Young Jun Chai, Sang Mok Lee, Su-jin Kim, June Young Choi, Kyu Eun Lee, Ki-Tae Hwang, Sun-Won Park, Ka Hee Yi

**Affiliations:** 1 Department of Surgery, Seoul Metropolitan Government Seoul National University Boramae Medical Center, Seoul, Korea; 2 Department of Surgery, Seoul National University Hospital and College of Medicine, Seoul, Korea; 3 Department of Surgery, Seoul National University Bundang Hospital, Gyeonggi-do, Korea; 4 Department of Radiology, Seoul National University College of Medicine, Seoul Metropolitan Government Seoul National University Boramae Medical Center, Seoul, Korea; 5 Department of Internal Medicine, Seoul Metropolitan Government Seoul National University Boramae Medical Center, Seoul, Korea; King Abdulaziz University Hospital, SAUDI ARABIA

## Abstract

The aim of this study was to evaluate preoperatively identifiable clinical and ultrasonographic characteristics associated with central lymph node metastasis (CLNM) in clinically node negative papillary thyroid carcinoma (PTC) patients. Records of the patients who underwent thyroidectomy with prophylactic central lymph node dissection due to clinically node negative PTC (size, 1.0–3.0 cm) were reviewed. Of a total of 174 patients, 71 (40.8%) had CLNMs. CLNM was more associated with capsule invasion than capsule non-invasion on ultrasonography (68.4% vs. 37.4%, p = 0.009). In the 155 patients without capsule invasion, a distance from the capsule < 1.9 mm was associated with CLNM in univariable (p = 0.002) and multivariable analysis (p < 0.001). Any PTC patient with a distance from the capsule ≥ 1.9 mm did not have CLNM whereas 40.8% (58/142) of PTC patients with a distance from the capsule < 1.9 mm had CLNM. CLNM was not associated with age, gender, or tumor size on ultrasonography. Distance from capsule ≥ 1.9 mm on preoperative ultrasonography was a significant indicator for not having CLNM in clinically node negative PTC patients. Measuring distance from the capsule on preoperative ultrasonography images could help select patients with PTC who could benefit from prophylactic central lymph node dissection.

## Introduction

Papillary thyroid carcinoma (PTC) is the most common endocrine malignancy worldwide, and its prognosis is usually favorable. However, some patients with PTC have a poor prognosis and develop local recurrence and distant metastases, and may die from the disease [1].The presence of central lymph node metastases (CLNM) is especially regarded as a risk factor for the persistence of disease, locoregional recurrence, distant metastases, and reduced survival rates [2, 3]. Therefore, preoperative detection of CLNM may potentially contribute to an improved prognosis for PTC.

Although ultrasonography is useful in the diagnosis of thyroid pathology, it is difficult to predict CLNM with ultrasonography. For the prediction of CLNM, the sensitivity of ultrasonography is low, with estimates of 38–59% [4–6], and a significant number of patients with CLNM are categorized into clinically node negative (cN0). Likewise, CLNM in these cN0 patients is common, ranging from 52 to 64% [7, 8]. In this regard, previous studies have attempted to predict CLNM by evaluating clinicopathological characteristics, rather than by detecting CLNM itself [5, 9, 10]. However, such risk factors for CLNM, which include extrathyroidal extension, multifocality, lymphovascular invasion, and genetic mutations, cannot be predicted preoperatively and are therefore of little use in determining surgical extent.

In this study, we investigated whether CLNM in cN0 PTC patients can be predicted by using preoperatively identifiable clinical and ultrasonographic features, including age, gender, tumor size, and the distance between the tumor and thyroid capsule.

## Patients and methods

The study was a retrospectively study and approved by the Institutional Review Board of Seoul Metropolitan Government Seoul National University Boramae Medical Center.

### Patients

Consecutive patients with solitary PTC (size, 1.0–3.0 cm) who underwent thyroidectomy combined with elective prophylactic central lymph node dissection (pCND) at Seoul National University Boramae Medical Center from January 2010 to December 2016 were eligible for this study. Tumors smaller than 1.0 cm or larger than 3.0 cm were excluded because pCND is not recommended for the tumors smaller than 1.0 cm, and routine pCND should be considered for the tumors larger than 3.0 cm. Medical records and ultrasonography images of patients were retrospectively reviewed. Preoperative ultrasonography was performed on all of the study patients. Ipsilateral pCND was performed routinely on the affected side and bilateral pCND was performed for PTCs in the isthmus. Patients were classified ascN0 when there was no evidence of central lymph node enlargement on ultrasonography or physical examination. To ensure an adequate pCND specimen, those patients with less than three CLNs were excluded. Patients with a variant of PTC, multifocal PTC, or lateral lymph node metastases were also excluded.

### Ultrasonography findings

All of the enrolled patients were investigated by ultrasonography (iU22 system, Philips, Seattle, WA, USA) within the 60 days before surgery. One clinician (C.Y.S.) and one neuroradiologist (S.W.P.) blinded to the clinical information independently retrospectively reviewed the preoperative ultrasonography images. When either the clinician or radiologist identified a case as having indefinite measurements, it was reviewed by both of them working together. The most recent ultrasonography record was selected if preoperative ultrasonography had been performed more than once.

**Fig 1:**
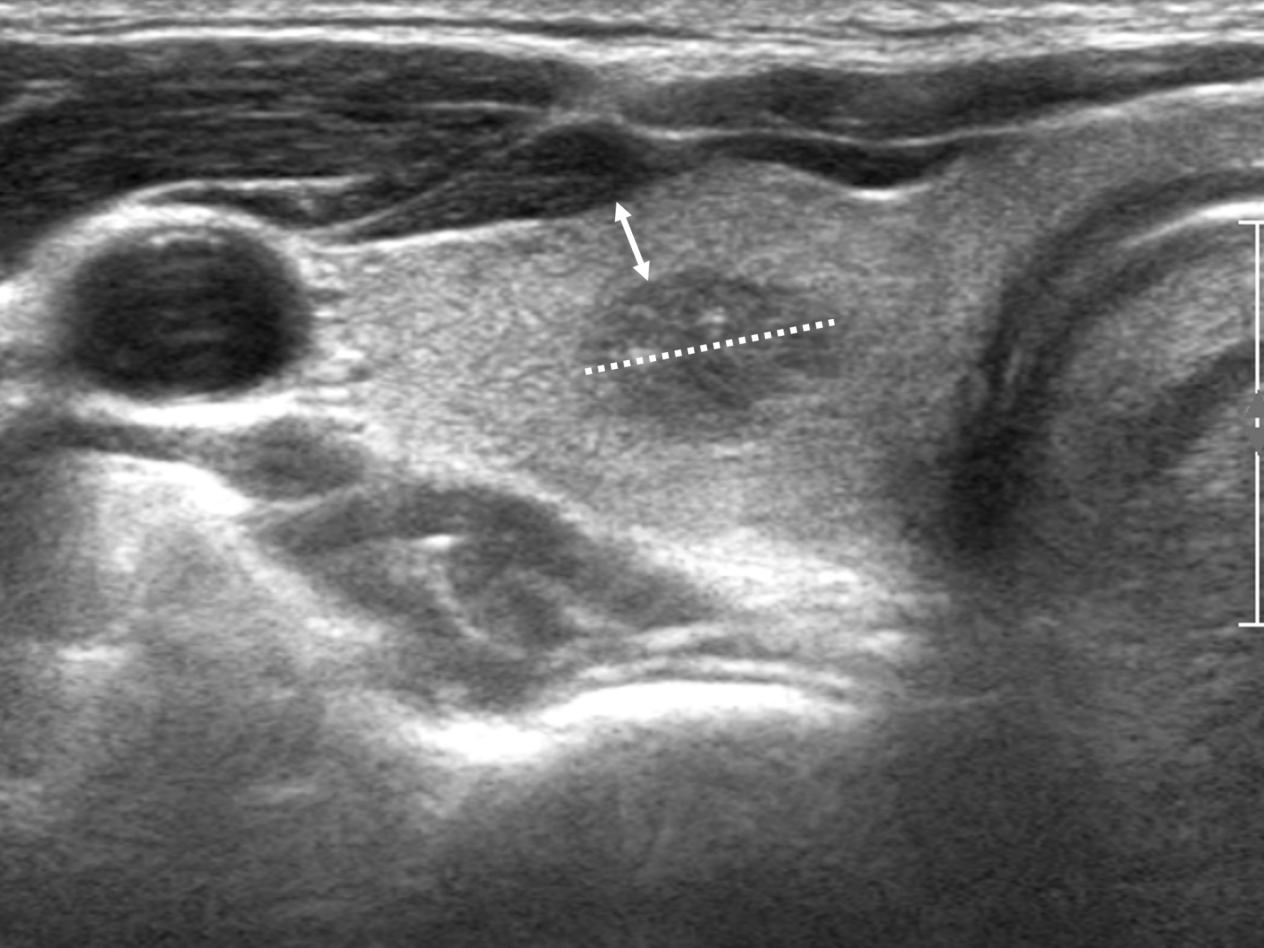
Tumor size and distance from capsule. Measurement of tumor size (dotted line) and distance from capsule (double headed arrow) on ultrasonography images.

The tumor size was defined as the longest diameter in three dimensions and the distance from the capsule was defined as the shortest distance from the tumor border to the thyroid capsule or trachea on transverse and longitudinal views (Fig 1). The distance from the capsule was recorded as 0 mm when the tumor abutted the thyroid capsule or trachea. The distance was recorded as *capsule invasion* when the tumor invaded the thyroid capsule or trachea on ultrasonography.

### Statistical analysis

Continuous variables were expressed as the mean with standard deviation. Categorical variables were compared using chi-square (*X*^2^) or Fisher’s exact tests. Dichotomous distribution of the data with the tumor size and the distance from capsule were chosen arbitrarily. Multivariable analysis was performed using binary logistic regression. The odds ratios (ORs) and 95% CI were reported for significant differences. A *p* value < 0.05 was considered to indicate statistical significance. The statistical analysis was performed using SPSS version 20 (IBM Corporation, Armonk, NY, USA).

## Results

### Clinicopathological characteristics

A total of 174 patients (145 females and 34 males) were enrolled in the study (Table 1). Seventy-one (40.8%) patients had CLNM. The mean age was 51.7 ± 14.1 years. The mean tumor size was 1.5 ± 0.5 cm (range, 1.0–3.0). The mean number of retrieved lymph nodes was 5.0 ± 4.0, and the mean number of metastatic lymph nodes in CLNM positive patients was 1.2 ± 2.3.

**Table 1:** Clinicopathological characteristics of the study patients.

	Total (n = 174)
Age, years	51.7 ± 14.1
<55	103 (59.2%)
≥ 55	71 (40.8%)
Gender	
Female	140 (80.5%)
Male	34 (19.5%)
Tumor size, cm	1.5 ± 0.5
Tumor location	
Right lobe	97 (55.7%)
Left lobe	69 (39.7%)
Isthmus	8 (4.6%)
Type of surgery	
Total thyroidectomy	105 (60.3%)
Thyroid lobectomy	69 (39.7%)
Number of retrieved lymph nodes (mean ± SD)	5.0 ± 4.0
Number of metastatic lymph nodes (mean ± SD)	1.2 ± 2.3
Central lymph nodes metastasis	
Absent	103 (59.2%)
Present	71 (40.8%)

### Association between central lymph node metastasis and tumor size or distance from capsule

**Fig 2:**
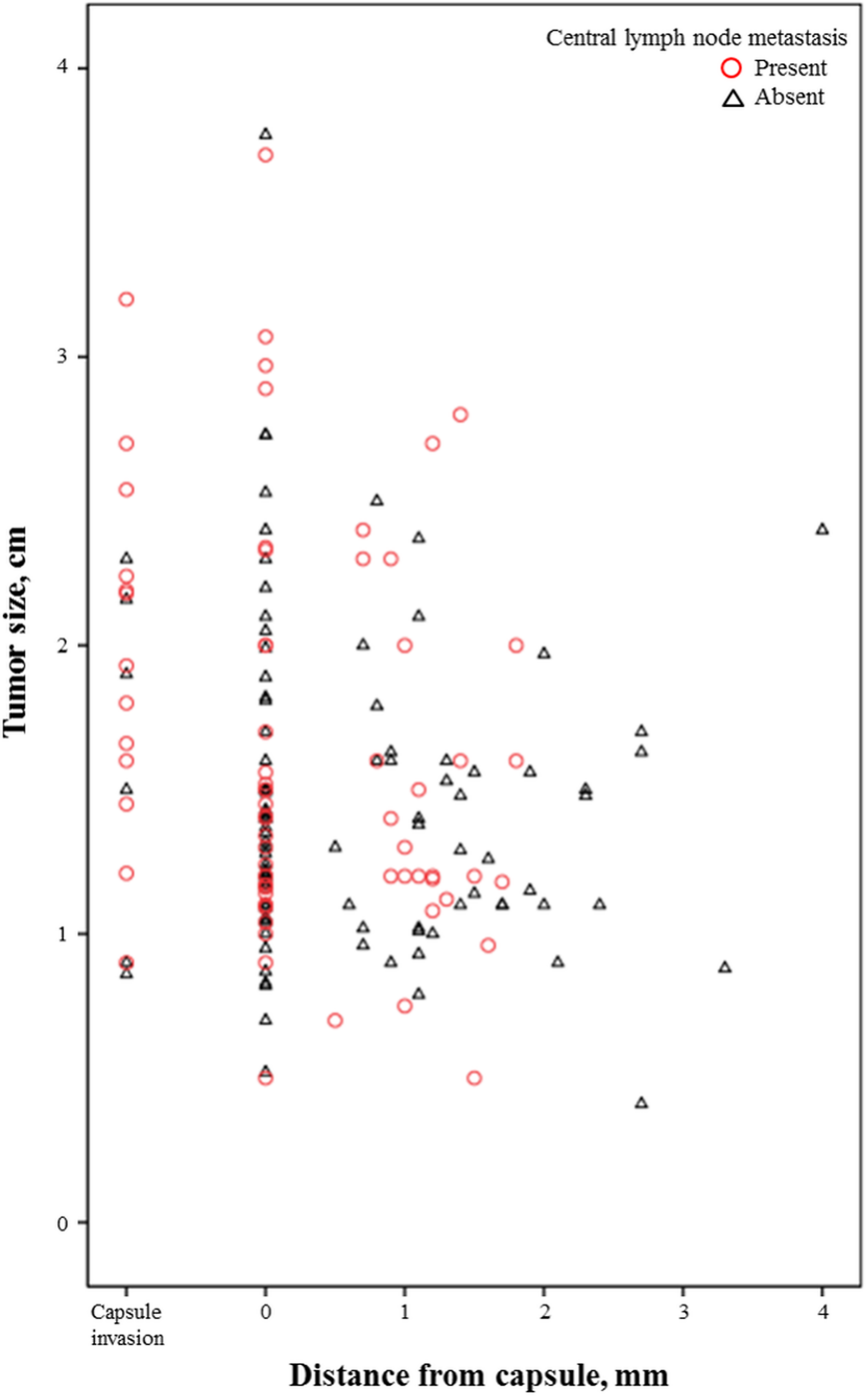
Tumor size and the distance from capsule on ultrasonography images. Tumors with capsule invasion are shown separately.

Tumor size and distances from the capsule on ultrasonography are shown in Fig 2. Tumor size on ultrasonography was not completely matched to the pathologic size, and ranged 0.4 cm to 3.7 cm. Tumors with capsule invasion are shown separately. There were 19 (10.9%) patients with capsule invasion on ultrasonography. Table 2 demonstrates that CLNM was more associated with capsule invasion than with capsule non-invasion (68.4% vs. 37.4%, p = 0.009). Excluding the 19 patients with capsule invasion on ultrasonography, the associations between CLNM and ultrasonographic findings were analyzed in 155 patients without capsule invasion (Table 3). In these patients, CLNM was not associated with age, gender or tumor size on ultrasonography. CLNM was more common in the PTC patients with a distance from the capsule < 1.9 mm (p = 0.002). Any PTC patient with a distance from the capsule ≥ 1.9 mm did not have CLNM whereas 40.8% (58/142) of PTC patients with a distance from the capsule < 1.9 mm had CLNM. In multivariable logistic regression analysis (Table 4), distance from the capsule < 1.9 mm was an independent risk factor of CLNM. Age, gender, or tumor size was not associated with CLNM.

**Table 2:** Central lymph nodal status according to capsule invasion on ultrasonography.

	Capsule invasion (-)	Capsule invasion (+)	*p* value
CLNM (-)	97 (62.6%)	6 (31.6%)	0.009
CLNM (+)	58 (37.4%)	13 (68.4%)	

**Table 3:** Central lymph nodal status of the tumor without capsule invasion according to age, gender, and ultrasonographic findings.

	CLNM negative (n = 97)	CLNM positive (n = 58)	*p* value
Age, years			
<55	51 (52.6%)	38 (65.5%)	0.133
≥ 55	46 (47.4%)	20 (34.5%)	
Gender			
Female	81 (83.5%)	45 (77.6%)	0.361
Male	16 (16.5%)	13 (22.4%)	
Tumor size on ultrasonography			
< 2 cm	83 (85.6%)	43 (74.1%)	0.077
≥ 2cm	14 (14.4%)	15 (25.9%)	
Distance from capsule			
< 1.9 mm	84 (86.6%)	58 (100.0%)	0.002
≥ 1.9 mm	13 (13.4%)	0 (0.0%)	

**Table 4:** Multivariate analysis of factors associated with central lymph node metastasis.

	Odds ratio	95% confidence interval	*p* value
Age ≥ 55	0.593	0.294–1.197	0.145
Male gender	1.599	0.666–3.842	0.294
Tumor size on ultrasonography ≥ 2cm	2.034	0.869–4.759	0.102
Distance from capsule< 1.9 mm	n.a^a^	n.a^a^	< 0.001

^a^ Not available.

## Discussion

ATA guidelines recommend the preoperative evaluation of neck lymph nodes by ultrasonography for all patients with biopsy-proven thyroid carcinoma [11]. The accuracy of ultrasonography in the detection of lateral neck lymph node metastases is well recognized [12].However, the sensitivity of ultrasonography for detecting CLNM in patients with PTC is low and variable [5, 12, 13]. This is because air in the trachea and complex structures in the clavicle and sternum may make it difficult for ultrasonography to detect CLNM.

Previous studies have reported several risk factors for CLNM in PTC patients such as an age younger than 55, male sex, large tumor size, extrathyroidal extension, multifocality, lymphovascular permeation, capsular invasion, and *BRAF*^V600E^ mutation [9, 14–16]. However, many of these risk factors are identified postoperatively. In this study, by contrast, we used only clinical and ultrasonographic findings to demonstrate risk factors for CLNM without using other additional modality requirements. It is notable that we used the distance between the tumor and capsule on ultrasonography to predict CLNM.

In fact, extrathyroidal extension is closely related to the presence of both capsular abutment and CLNM [17, 18]. Therefore, it was reasonable to speculate that an association exists between the distance from capsule and CLNM, which was shown to be the case in this study. Tumor location on the thyroid capsule was also emphasized in an observational trial conducted in Japan [19]. In that study, tumor location on the surface of the thyroid or adjacent to the trachea was considered an unfavorable feature, and the patients with this feature were excluded from the observational trial [19]. In the present study, any PTC patient with a distance from the capsule≥ 1.9 mm on ultrasonography did not have CLNM, suggesting that a distant location of the PTC from the capsule is a strong indicator of the absence of CLNM. The results suggest that pCND is unnecessary in cN0 PTC patients when the distance from the capsule is ≥ 1.9 mm.

Tumor size is well known as a risk factor for CLNM, and previous studies have reported thresholds for tumor size associated with CLNM. A tumor size of 0.5 to 2cm was shown to be associated with occult CLNM in the studies, but the incidence of CLNM in cases with a tumor size smaller than the suggested threshold was still considerable, ranging from 26–55% [7, 20, 21]. Likewise, 19.1% (18/94) of patients with a tumor size < 8 mm still had CNLM in this study, although a tumor size ≥ 8 mm was identified as an independent risk factor for CLNM. This result suggests that one should be careful in using tumor size to predict CLNM.

In this study, the rate of occult CLNM was 40.8%. The role of pCND in cN0 PTC patients is controversial, with the current ATA guidelines not recommending routine pCND [22].The reason for this is that although performing pCND could detect occult CLNM, it has not been associated with improvement in recurrence or survival rates [23–25]. Additionally, patients undergoing central compartment neck dissection have been shown to suffer greater rates of morbidity, such as hypocalcemia and vocal cord paralysis, and there was also a cost-effectiveness disadvantage in comparison to patients who underwent total thyroidectomy alone [25–27]. Thus, a more selective approach is necessary to determine the requirement for pCND in patients with PTC. According to the results of this study, occult CLNM was associated with a distance from the capsule < 1.9 mm. Succinctly, patients did not harbor CLNM when the distance from the capsule was ≥ 1.9 mm, indicating that pCND should not be considered in such patients.

This study has limitations because of its retrospective design. First, the ultrasonographic images were retrospectively reviewed; thus, there is the possibility that tumor size and distance from the capsule measured in the study were different from the actual size and distance. However, tumor images in which the tumor is the largest are usually saved as representative images, and the distance from the capsule is usually the shortest in the representative image when the tumor is seen to be the largest. Therefore, we believe that the size and the distance measured on the ultrasonography images were almost the same as the actual tumor size and the distance, although we do believe prospective study is necessary to evaluate the distance correctly. Second, the number of the nodules investigated was relatively small; thus variables such as location and combined thyroiditis could not be analyzed. Third, the criteria 1.9 mm is not easy to use in the clinical practice. More practical distance should be found in the future studies. Further studies with larger number of nodules in multiple institutes are necessary to suggest more easily applicable criteria.

## Conclusions

In conclusion, a distance from the capsule < 1.9 mm was associated with CLNM in cN0 PTC patients. This finding may be helpful for surgeons making the decision on whether to perform pCND in such patients. Further prospective studies with a larger number of patients might be necessary.
